# Roles of Spatial Parameters on the Oscillation of Nuclear NF-κB: Computer Simulations of a 3D Spherical Cell

**DOI:** 10.1371/journal.pone.0046911

**Published:** 2012-10-03

**Authors:** Daisuke Ohshima, Jun-ichiro Inoue, Kazuhisa Ichikawa

**Affiliations:** 1 Division of Mathematical Oncology, The Institute of Medical Science, The University of Tokyo, Minato-ku, Tokyo, Japan; 2 Division of Cellular and Molecular Biology, The Institute of Medical Science, The University of Tokyo, Minato-ku, Tokyo, Japan; Rutgers University, United States of America

## Abstract

Transcription factor NF-κB resides in the cytoplasm and translocates to the nucleus by application of extracellular stimuli. It is known that the nuclear NF-κB oscillates and different oscillation patterns lead to different gene expression. Nearly forty reports on modeling and simulation of nuclear NF-κB have been published to date. The computational models reported so far are temporal or two-dimensional, and the discussions on spatial parameters have not been involved or limited. Since spatial parameters in cancer cells such as nuclear to cytoplasmic volume (N/C) ratio are different from normal cells, it is important to understand the relationship between oscillation patterns and spatial parameters. Here we report simulations of a 3D computational model for the oscillation of nuclear NF-κB using A-Cell software. First, we found that the default biochemical kinetic constants used in the temporal model cannot replicate the experimentally observed oscillation in the 3D model. Thus, the default parameters should be changed in the 3D model. Second, spatial parameters such as N/C ratio, nuclear transport, diffusion coefficients, and the location of IκB synthesis were found to alter the oscillation pattern. Third, among them, larger N/C ratios resulted in persistent oscillation of nuclear NF-κB, and larger nuclear transport resulted in faster oscillation frequency. Our simulation results suggest that the changes in spatial parameters seen in cancer cells is one possible mechanism for alteration in the oscillation pattern of nuclear NF-κB and lead to the altered gene expression in these cells.

## Introduction

The activation of the transcription factor NF-κB leads to a wide range of cellular responses including proliferation, apoptosis, and angiogenesis. More than 500 genes have been reported to be expressed upon activation of NF-κB including the immune-responsive and NF-κB regulatory genes in addition to proliferation-, invasion/metastasis- and angiogenesis-promoting genes [1], [2], [3], [4], [5], [6]. While NF-κB activation in normal cells is mostly transient, it is constitutively activated in malignant tumors and stimulates the growth of malignant cells [1], [7], [8]. Thus, the control of NF-κB activity is critical in cancer therapies.

NF-κB is activated through two main pathways known as the classical (canonical) and the non-classical (non-canonical) pathways. In the classical pathway, NF-κB is activated by TNFα, IL1β, or bacterial products [3], [4], [7], [9], [10], [11], [12], [13], [14], [15], [16]. IL-1 stimulation results in the formation of a signaling complex composed of TRAF6, TAK1, and MEKK3 [17] which leads to the activation of TAK1 and MEKK3 [18]. IKK complex, which is a heterotrimer of IKKα, IKKβ, and NEMO (IKKγ) in the classical pathway, is recruited to the complex, and NEMO is ubiquitinated leading to the activation of IKK [19]. Activated IKK then phosphorylates IκBα in the NF-κB complex, which is a heterotrimer of IκBα, p50, and p65 (RelA) [20], [21]. The phosphorylated IκBα is subsequently ubiquitinated and subjects to proteasomal degradation leading to the release of inhibition on NF-κB by IκBα [22]. Thus activated NF-κB translocates to the nucleus, where it binds to the promoter or enhancer region of target genes.

Interestingly, the concentration of nuclear NF-κB is known to oscillate by the application of TNFα. The analysis of a population of cells showed damped oscillation of nuclear NF-κB with a period of 1.5–3 hrs [17], [23]. Damped oscillation of NF-κB was also reported in a single cell analysis with a period of 1–2 hrs using RelA fused to red fluorescent protein [24], [25]. It has been reported that changes in the oscillation pattern of nuclear NF-κB led to changes in the gene expression pattern. Hoffmann et al. reported that shorter and longer applications of TNFα resulted in non-oscillating and oscillating nuclear NF-κB, respectively, and this difference led to the expression of quick and slow responsive genes [23]. It has also been reported that the change in the oscillation frequency, which was mimicked by changing the interval of pulsatile TNFα stimulation, resulted in different gene expression patterns [24]. Thus, it is thought that the oscillation pattern of nuclear NF-κB is important to the selection of expressed genes [24], [26], [27].

According to experimental observations on the oscillation of nuclear NF-κB, nearly 40 computational models have been published. Among them, a model by Hoffmann et al. was the first to show the oscillation of nuclear NF-κB in computer simulation [23]. Their computational model included continuous activation of IKK, degradation of IκBα, shuttling of NF-κB between the cytoplasm and nucleus, and NF-κB-dependent gene expression and protein synthesis of IκBα. Their simulations showed good agreement with experimental observations. After Hoffmann's model, many models have been published showing the effect of A20, a negative regulator of NF-κB [28], IκBε or IκBδ, other inhibitors of NF-κB [29], [30], phosphorylation and dephosphorylation of IKK [31], [32], and IKK-dependent and independent degradation pathways for IκBα [33]. Characterization of oscillation [25], [32], [34], [35], [36], [37] and sources of cell-to-cell variability of oscillation [25], [37], [38], [39], [40], [41] were also reported. Recently, a possible role of the oscillation of nuclear NF-κB as the decision maker for the cell fate by counting the number of oscillations was proposed [27]. None of these models are complicated, yet it is not easy to explain the essential mechanism of oscillation. There is a report on simplified computational models showing the minimal components of the oscillation of nuclear NF-κB [42]. This analysis showed essentially the same mechanism of oscillation that was reported previously in more abstracted forms [43], [44].

Thus the oscillation of nuclear NF-κB is a good example of collaboration between *in vitro* and *in silico* experiments. However, all computational models shown above are temporal models and include no discussion on spatial parameters such as diffusion coefficient, nuclear to cytoplasmic volume (N/C) ratio, nor the location of protein synthesis within the cytoplasmic compartment. In contrast to these temporal models, a two-dimensional model was published showing that changes in the geometry of the nucleus altered the oscillation pattern of nuclear NF-κB [45]. However, a three-dimensional (3D) model is important to compare its simulation results reasonably with observations. Here we construct a 3D model, and investigate the oscillation patterns of nuclear NF-κB by changing spatial parameters. First we find that the parameters used in the temporal model must be changed in the 3D model to obtain the observed oscillation pattern. Second, spatial parameters strongly influence oscillation patterns. Third, among them, N/C ratio strongly influences the oscillation pattern. Fourth, nuclear transport, which would be changed by the increase or decrease of nuclear pore complexes (NPCs), also has a strong effect on changes in the oscillation pattern. In summary, our simulation results show that changes in spatial parameters such as the N/C ratio result in altered oscillation pattern of NF-κB, and spatial parameters, therefore, will be important determinants of gene expression.

## Results

### Temporal model reproduces an observed oscillation

**Figure 1:**
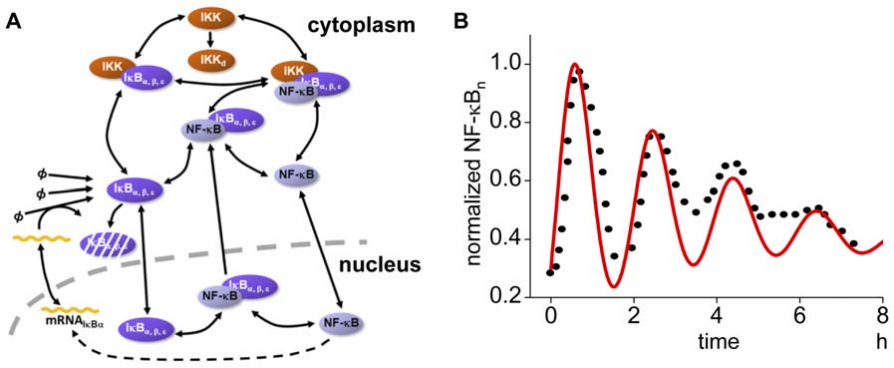
Schematic view of the temporal model and its simulation result. (A) The model includes IKK activation, subsequent phosphorylation and proteosomal degradation of inhibitory protein IκBα, IκBβ, and IκBε, activation of NF-κB, and its translocation to nucleus where a gene for IκBα is expressed in the NF-κB-dependent manner. (B) The simulated oscillation of the temporal model (red line) and an experimental observation by Sung, M.L. *et al*., PLos ONE, 2009 [25] (dots) are shown. The concentration of nuclear NF-κB (NF-κB_n_) is normalized to the maximum value.

We began with a temporal model comparing simulation results with the published oscillation pattern in a single cell. Our model includes the degradation of IκBs (i.e. IκBα, IκBβ, and IκBε) by activated IKK, subsequent activation and translocation of NF-κB to the nucleus, and gene expression and protein synthesis of IκBα (Figure 1A, and Materials and Methods for detail). The simulated nuclear NF-κB concentration (NF-κB_n_, red line in Figure 1B, which is normalized to the maximum value) agrees with a typical experimental observation (dots in Figure 1B) [25]. Parameter values for this simulation are shown in Table S1.

### Rate constants in the temporal model do not reproduce the same oscillation pattern in the 3D model

**Figure 2:**
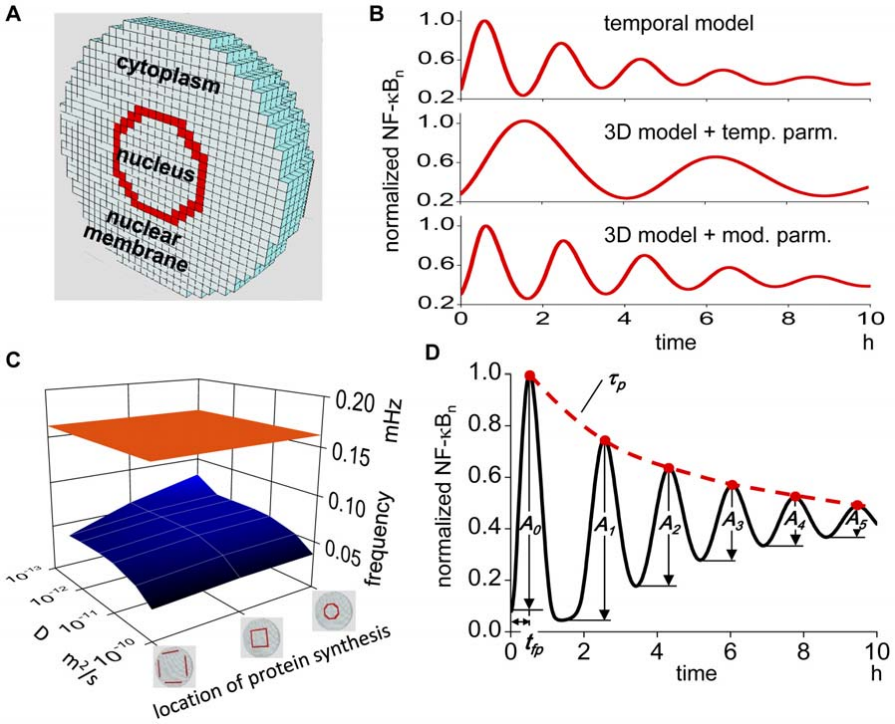
3D model requires a different parameter set from that used in the temporal model. (A) 3D model of spherical cell with diameter of 50 µm, which is divided into compartments enabling reaction-diffusion simulation. Red compartments indicate the nuclear membrane compartments. (B) Middle panel is the 3D simulation result with the same reaction rate constants as in the temporal model. The simulation result shows much lower oscillation frequency as compared to the temporal model shown in the top panel. Bottom panel is the oscillation in the 3D simulation with modified reaction rate constants. (C) No combination of diffusion coefficient and the location of IκBs protein synthesis (blue plane) gives comparable oscillation frequency as in the temporal model (orange plane). The range of D is 10^−13^ to 10^−10^ m^2^/s with three locations of IκBs protein synthesis, which are indicated by three icons. (D) We defined oscillation frequency *f*, height of the first peak *A_0_*, time to the first peak *t_fp_*, decay time constant of the peak *τ_p_*, and decay time constant *τ_d_* of successive amplitudes *A_0_*, *A_1_*, *A_2_*…., as parameters characterizing nuclear NF-κB oscillation.

Next, we constructed a spherical 3D cell model with a diameter of 50 µm, which is divided into cubic compartments with identical edge length of 1.52 µm to allow reaction-diffusion simulations in 3D space (Figure 2A). The compartments were divided into three regions: the cytoplasm, nucleus, and nuclear membrane. The same reaction schemes used in the temporal model were embedded in the corresponding compartments in the 3D model (see Materials and Methods for more detail). The diffusion coefficients of NF-κB, IKK complex, and IκBs are not known; we employed 10^−11^ m^2^/s, which is in the range of soluble proteins [46], [47], [48], [49], [50]. The diffusion coefficient for mRNA was 10^−13^ m^2^/s [51]. An N/C ratio of 8.3% was employed [52], [53]. As the intracellular location of protein synthesis of IκBs is not known, we selected the same compartments as the nuclear membrane. These conditions: the diffusion coefficients of 10^−11^ and 10^−13^ m^2^/s for proteins and mRNA respectively, an N/C ration of 8.3%, and IκBs synthesis on the compartments of nuclear membrane, are referred to as the canonical spatial conditions. These conditions will be changed to investigate their effects on the oscillation in the following section. We analyzed the simulated oscillation of NF-κB_n_ at the most peripheral compartment of the nucleus because the spatial heterogeneity of NF-κB_n_ was negligible in our simulations (Figure S1).

First, we ran simulation using the same rate constants as in the temporal model. The simulated oscillation in the 3D model (Figure 2B, middle) shows much lower frequency in comparison to the temporal model (Figure 2B, top). Thus, the oscillation does not agree with an experimental observation using the same rate constants as in the temporal model.

There is a possibility that the oscillation pattern in the 3D model might agree with the temporal observation if we selected some combination of spatial parameters. Therefore, we ran simulations changing canonical spatial conditions within the range of diffusion coefficient of proteins from 10^−10^ to 10^−13^ m^2^/s and three locations of protein synthesis, which are shown by red compartments if Figure 2C. The N/C ratio was not changed because the value was reported to remain constant irrespective of cell size [52], [54]. The oscillation frequency was calculated from the distance between the first and the second peaks. Simulation results showed that any combinatorial changes of these spatial parameters were unable to generate an oscillation frequency that agrees with the temporal observation (Figure 2C).

These simulation results indicate that rate constants used in the temporal model should be changed in the spherical 3D cell model. To determine which rate constants could duplicate the observed temporal oscillation, we ran a set of simulations and found rate constants with which the oscillation pattern in the 3D model fitted the experimental observation (Figure 2B, bottom). The selected set of rate constants shown in Table S2 is the basis for the following analysis and is referred to as the control temporal conditions. The combination of canonical spatial and control temporal conditions is simply referred to as the control conditions. A movie of the oscillation of NF-κB in the control condition is available (Video S1).

### Oscillation pattern is characterized quantitatively by five parameters

To evaluate the oscillation pattern quantitatively, we defined five parameters that characterize oscillations called the characterizing parameters. They are 1) frequency (*f*), 2) amplitude of the first peak (*A_0_*), 3) time to the first peak (*t_fp_*), 4) decay time constant for the peaks in a oscillation (*τ_p_*), and 5) decay time constant *τ_d_* of the successive amplitudes (i.e. *A_0_*, *A_1_*, **…**) (Figure 2D). The frequency was obtained by Fourier analysis. Amplitude was normalized to the maximum peak value of NF-κB_n_ at the control conditions. Parameters *τ_p_* and *τ_d_* are measures of persistency of oscillation. Their larger values indicate longer-lasting oscillation. Several of these parameters were analyzed in the temporal model [35], [36]. In the control conditions, *f*, *t_fp_*, *τ_p_*, and *τ_d_* are 0.139 mHz, 0.617 hrs, 9.32 hrs, and 7.14 hrs, respectively.

### N/C ratio alters the oscillation pattern

It is reported that in human cancer patients, both nuclear volume and N/C ratio are increased [52], [55], and more importantly, they are positively correlated with the progression and malignancy of the cancer [56], [57], [58], [59]. Hence, it is important to determine if the oscillation pattern changes with N/C ratio changes.

**Figure 3:**
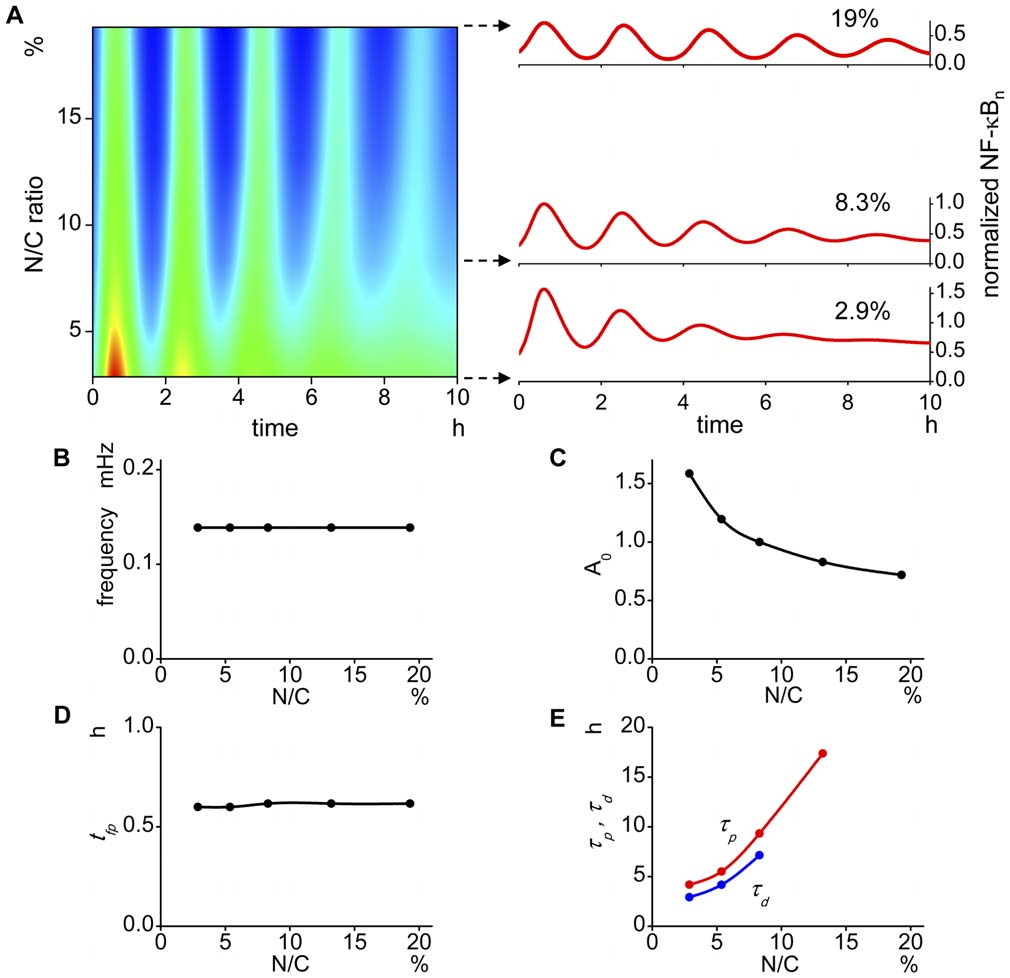
The oscillation pattern is altered by the change in N/C ratios. (A) Oscillation time courses are plotted for varying N/C ratios from 2.9 to 19% with the amplitude shown in color for higher and lower in red and blue, respectively. Representative oscillations are shown on the right. This representation shows overall oscillation pattern. There is no change in the oscillation frequency by changing N/C ratios which is seen by a regular color interval among different N/C ratios. The damping of the oscillation is faster in smaller N/C volume ratios which is supported by disappearance of the periodic color change at the later time in smaller N/C ratios. At higher N/C ratios, however, the oscillation lasts for more than 10 hrs. (B) There is no change in the oscillation frequency (*f*) with changes in the N/C ratio. (C) The amplitude of the first peak (*A_0_*) becomes smaller at larger N/C values. (D) The time to the first peak (*t_fp_*) also stays almost unchanged by the change in N/C. (E) The decay time constants of the peaks (*τ_p_*) and successive amplitudes (*τ_d_*) of oscillation becomes larger at larger N/C ratios. *τ_p_* and *τ_d_* at larger N/Cs could not be extracted from the simulated oscillation.

We summarized all oscillations tested for N/C ratios from 2.9 to 19% along a time from 0 to 10 hrs with amplitudes in red and blue for higher and lower NF-κB_n_, respectively, together with ordinary plots of time courses at N/C ratios of 2.9, 8.3 (control), and 19% (Figure 3A). This representation tells us overall alteration of oscillation pattern by changes in N/C ratio. It is clearly seen that the oscillation frequency remains largely unchanged by changes in N/C ratio because the intervals of the color changes along the horizontal axis are almost the same for all N/C values tested. This is also shown by Fourier analysis (Figure 3B). There is no significant change in *t_fp_*, either because the time to the first peak (reddish, yellowish or greenish color depending on N/C ratio) does not change much in Figure 3A and is quantitatively shown by the lack of change in *t_fp_* (Figure 3D). However, there is a large change in *A_0_*, being smaller for larger N/C ratios, which is shown in the color change at the first peak from red to green from smaller to larger N/C ratios. This change is quantitatively shown (Figure 3C). The change in the persistency of oscillation is also seen by changes in N/C ratios. At an N/C ratio of 2.9%, the color change along the time axis disappeared around at 6 hrs; after this time, the color stays green, indicating cessation of oscillation. At a larger N/C ratio of 19%, however, the periodic color change continues for more than 10 hrs indicating prolonged oscillation. These are shown quantitatively by the changes in *t_p_* and *τ_d_* (Figure 3E). We cannot determine *τ_p_* and *τ_d_* at higher N/C ratios because the decays are not fitted with an exponential curve. Some plots are interrupted in the later figures for the same reason or a limited number of points in our simulation.

The results indicate that the oscillation pattern is altered greatly by changes in N/C ratios. In our simulation, the smaller N/C ratios result in damped oscillation, which can be compared with the preceding study showing suppressed oscillation by reduction in the nuclear radius in the 2D model [45].

### Rate of nuclear transport alters the oscillation pattern

**Figure 4:**
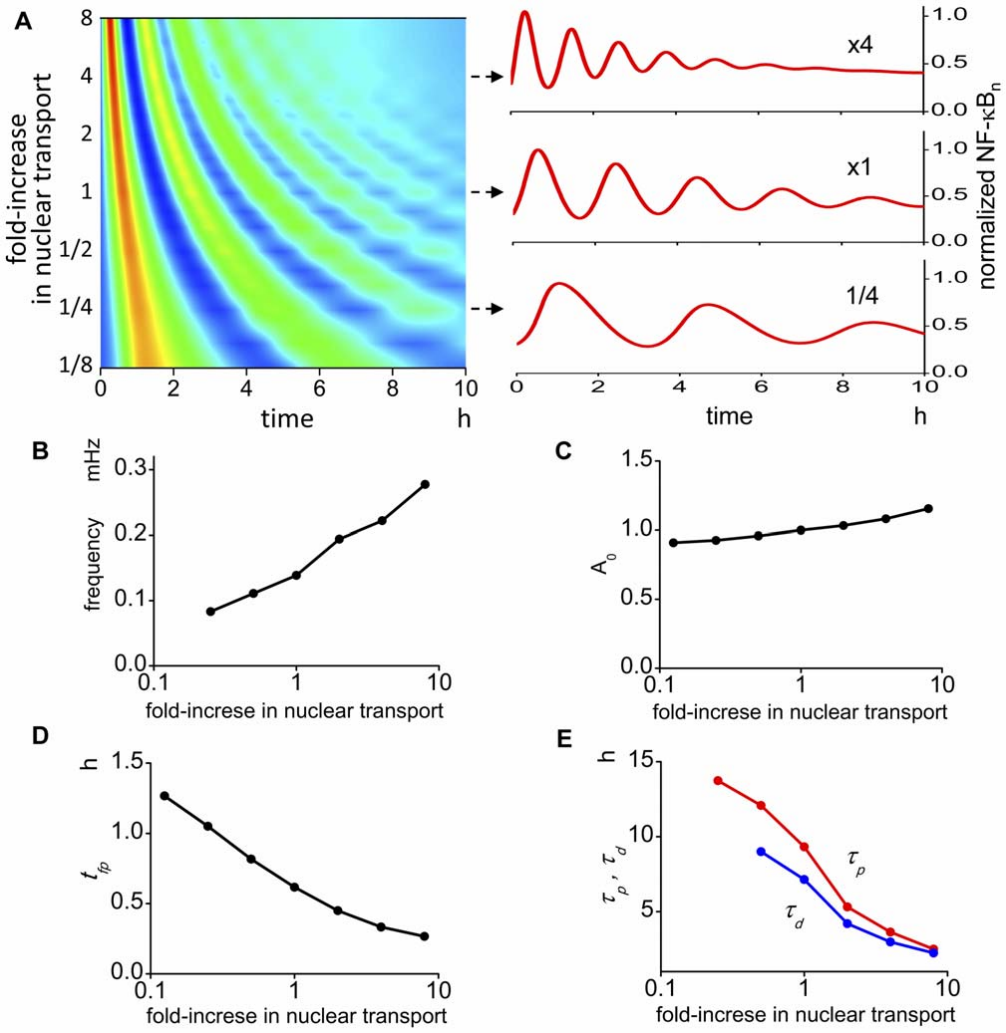
The oscillation pattern is altered by the change in the nuclear transport. (A) There is a large change in the oscillation pattern by the change in the nuclear transport. Representative oscillations are shown in the right panels. (B) Oscillation frequency becomes larger as transport increases. (C) The amplitude of the first peak becomes larger at larger transport values. (D) The time to the first peak changed largely by the change in the transport. (E) *τ_p_* and *τ_d_* also show large change by the change in the transport. These data are not available at smaller nuclear transport values.

There are reports suggesting an increase in NPCs in cancer cells leads to an increased nuclear transport [60], [61], and in addition, nuclear transport will be increased by the larger surface area of the nucleus that is seen in cancer cells. Hence, it is important to know if the oscillation pattern is modified by changes in nuclear transport. The summary shows that the change in the oscillation pattern due to changes in the nuclear transport is different from that seen in changes of the N/C ratio (Figure 4A). By changing the nuclear transport, all characterizing parameters are altered. Changes in *f* and *A_0_* are positively correlated with nuclear transport (Figure 4B and C). In contrast, *t_fp_*, *τ_d_*, and *τ_p_* are negatively correlated with increasing nuclear transport (Figure 4 D and E).

We also ran simulations by changing inward or outward nuclear transport separately. The results show large changes in the oscillation pattern. In addition, the oscillation change is not simple but shows biphasic alterations (Figure S2). In summary, the change in the nuclear transport altered *f*, *t_fp_*, *τ_p_* and *τ_d_* greatly, while the change in *A_0_* is not large.

### Diffusion coefficient alters the oscillation pattern

**Figure 5:**
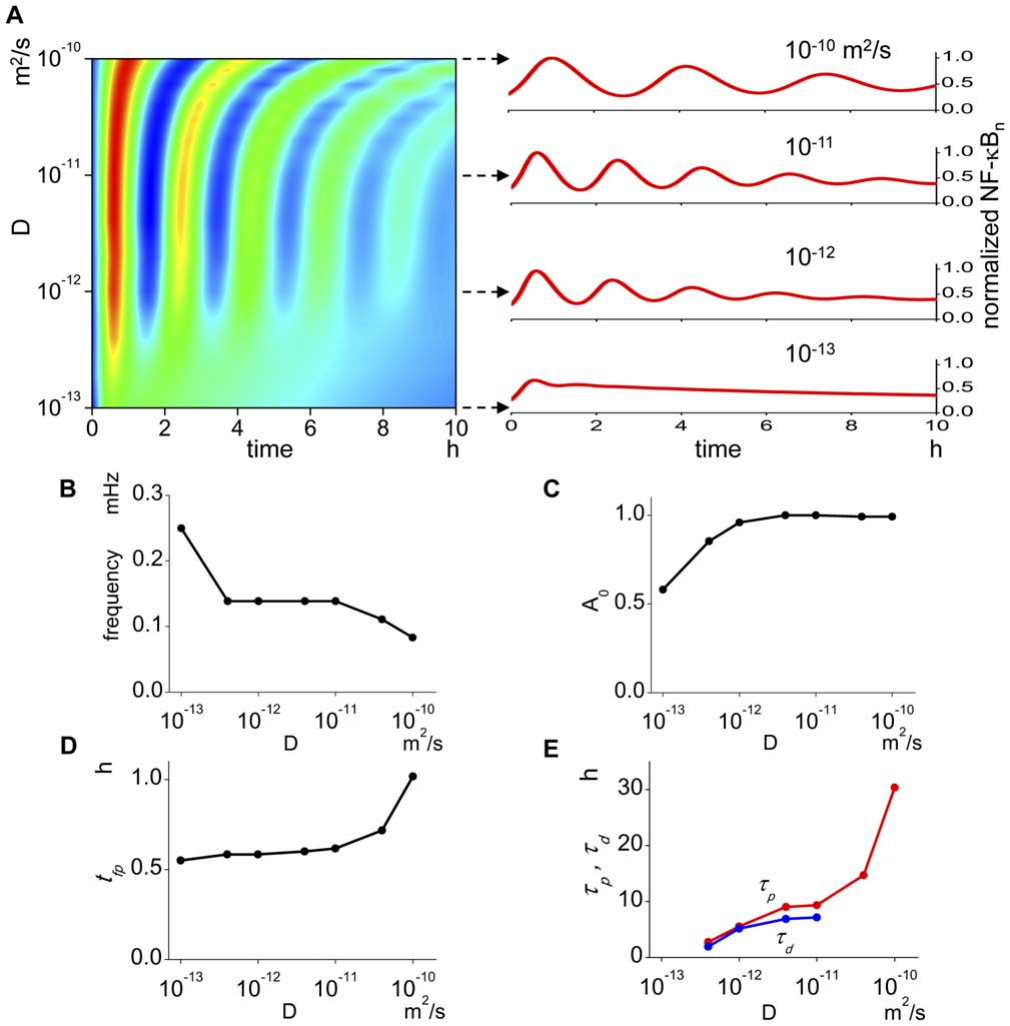
The oscillation pattern is affected by the change in the diffusion coefficient. (A) There are large changes in the oscillation pattern at low and high diffusion coefficients. At low diffusion coefficients, virtually no oscillation is seen. Right panels show representative oscillations. (B) At diffusion coefficients from 10^−12^ to 10^−11^ m^2^/s, the oscillation frequency stays unchanged. At higher diffusion coefficient, however, the oscillation frequency becomes lower, and at lower diffusion coefficient, it becomes higher. (C) At diffusion coefficient lower than 10^−12^ m^2^/s, the amplitude of the first peak increases as the diffusion coefficient increases. But it stays unchanged at higher diffusion coefficients. (D) The time to the first peak stays unchanged until the diffusion coefficient is increased to 10^−11^ m^2^/s, and at higher diffusion coefficient, it increases drastically. (E) *τ_p_* and *τ_d_* increase by the increases in the diffusion coefficient.

The diffusion coefficient is thought to be inherent to each protein. However, its effective value will be changed by changes in the density, volume, or surface area of the mitochondria, ER, and other organelles. The diffusion coefficient shows significant effect on the oscillation pattern (Figure 5A). While *f* stays unchanged with D in the middle range, it is increased or decreased at lower or higher values outside this range (Figure 5B). *A_0_* increases with increases in D until D reaches 10^−12^ m^2^/s and stays almost unchanged at larger values (Figure 5C). The parameter *t_fp_* stays almost unchanged at lower D, then increases abruptly at higher Ds (Figure 5D). Both *τ_d_* and *τ_p_* become larger with increasing D (Figure 5E). Thus, larger values of D result in prolonged oscillation. In summary, the diffusion coefficient affects the oscillation pattern significantly but differently from N/C ratio and nuclear transport.

### The location of IκB synthesis alters the oscillation pattern

**Figure 6:**
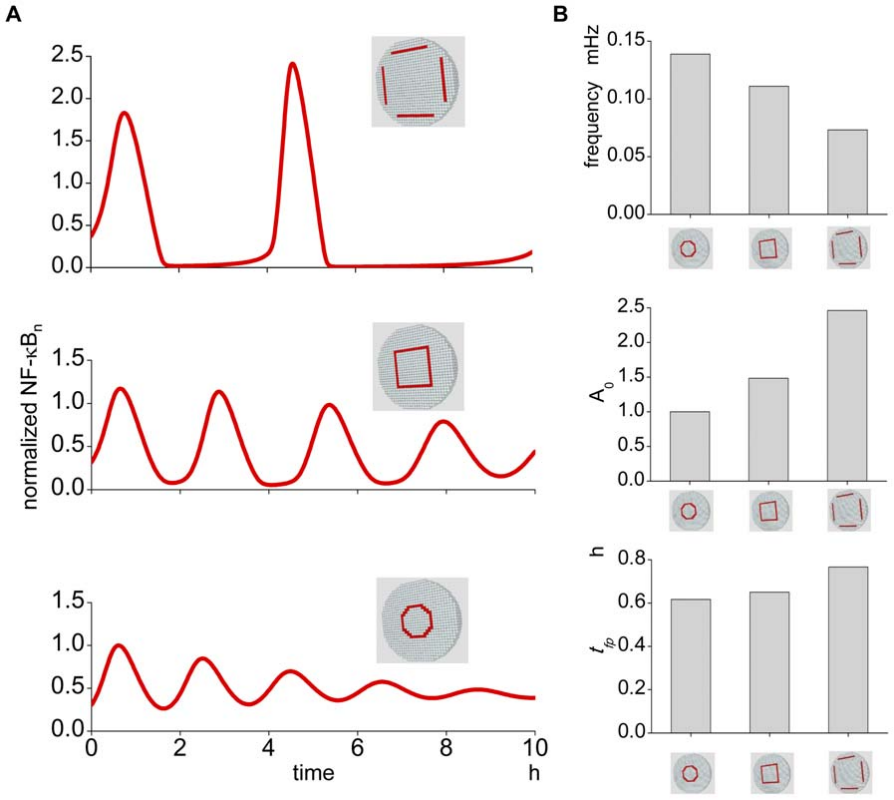
The oscillation pattern is changed by the change in the location of the IκBs synthesis. (A) The IκBs synthesis proceeds in the same compartment as the nuclear membrane in the control condition (bottom panel). If its location is moved to the plasma membrane keeping other parameters unchanged, the oscillation pattern is changed (middle and top panels). The loci of IκBs synthesis are shown in red compartments. (B) The changes in the oscillation frequency, amplitude of the first peak, and the time to the first peak are quantitatively shown in the top, middle, and bottom panels, respectively.

IκBs are the important determinants of the oscillation pattern of NF-κB_n_ [29], [42]. However, the exact intracellular location of their syntheses is not known. Then, we ran simulations to see the effect of changing synthesis locations. The location of IκBs syntheses at the control conditions is at the nuclear membrane compartments. We changed this location to the middle and the distant locations from the nuclear membrane while keeping the amount of IκBs syntheses constant. The alteration of oscillation pattern was greater than we expected (Figure 6A); *f* decreases and *A_0_* and *t_fp_* increases, respectively, as the synthesis is more distant from the nuclear membrane (Figure 6B). These simulation results indicate that the location of IκBs syntheses is also an important determinant for the NF-κB_n_ oscillation pattern.

### The location of transcription in a nucleus does not alter the oscillation pattern

In the simulations described thus far, transcription was assumed to occur uniformly within the nucleus. If we take a time-averaged location of a specific gene, it may distribute nearly uniformly within the nucleus. However, at some time point, a specific gene should be located somewhere in a nucleus, and more importantly, it has been suggested that the spatial fluctuation of the genome is not perfectly random but possesses some ‘territory’ [62]. Therefore, we ran simulations to see the effect of localized transcription in a nucleus. The center compartment of the nucleus was selected for the localized transcription of IκBs as the opposite extreme case from the control conditions. The rate of transcription was kept unchanged from the spatially integrated value in the control conditions. The simulation shows virtually no difference in the oscillation pattern by this localized transcription of IκBs (Figure S3). Thus, the oscillation pattern is not altered by the change in the locus of IκBs transcription.

### Localized IKK activation does not alter the oscillation pattern

In the control condition, IKK is activated in all cytoplasmic compartments. However, if we changed this global spatial condition to localized IKK activation, the oscillation pattern might change. We ran simulations that kept the spatially integrated rate of IKK activation unchanged but changed the locus of activation. Unexpectedly, we cannot see any change in the oscillation pattern (Figure S4). Even in the most extreme cases where IKK is activated at a single plasma-membrane compartment (Figure S4A, middle), the oscillations almost perfectly overlapped to the control conditions. Thus, the locus and distribution of IKK activation do not change the oscillation pattern in our simulation conditions.

### Characterizing parameter has different sensitivities to different spatial parameters

**Figure 7:**
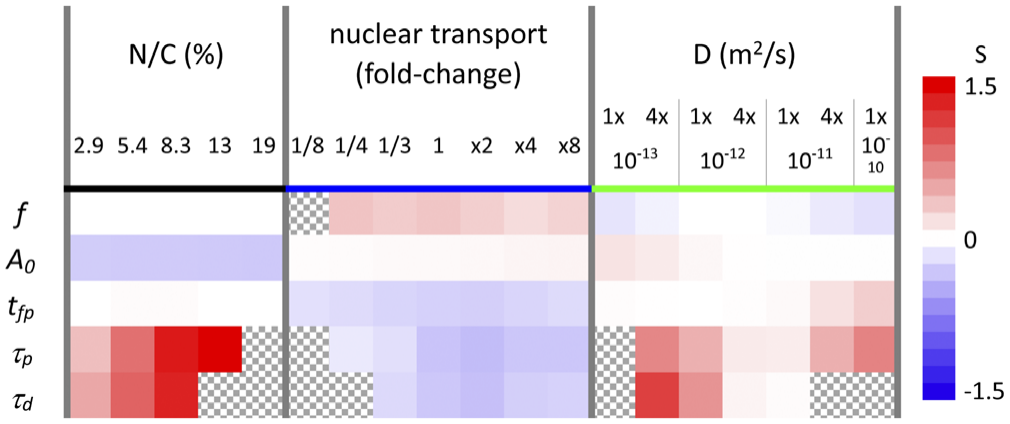
Sensitivity of each characterizing parameter by the change in the spatial parameters. Sensitivities for the five characterizing parameters of *f*, *A_0_*, *t_fp_*, *τ_p_*, and *τ_d_* to each spatial parameters, which are calculated by Eq.1, are shown (see Materials and Methods). Red and blue cells indicate positively and negatively large sensitivities, while white cells indicate that the characterizing parameter is not sensitive to the change in the corresponding spatial parameter. Hatched regions indicate no available data.

Sensitivity analysis is a valuable analytical method to see the effectiveness of parameter changes on the phenomenon of interest [63]. We performed sensitivity analysis at several points on N/C ratio, nuclear transport, and D (Figure 7A). Positive and negative sensitivities are shown in reddish and bluish colors, respectively, with deeper colors for larger sensitivities. The numbers shown on the right to the color bar are sensitivities calculated by Eq. 1 (see Materials and Methods). Hatched regions indicate no available data.

It is clearly seen that all characterizing parameters possess different sensitivities to different spatial parameters. For example, *f* possesses positive and negative sensitivity to nuclear transport and D, respectively, while it is insensitive to N/C ratios. On the other hand, *A_0_* are negatively sensitive to N/C ratio. Its sensitivity to nuclear transport and D is slightly positive. The sensitivity of the first peak *t_fp_* to N/C ratio is slightly positive, and *τ_p_* and *τ_d_* have the same tendency toward positive or negative sensitivity to the same spatial parameters. It is also clearly seen that each characterizing parameter possesses insensitive regions within a certain range of spatial parameters. For example, *f* is insensitive within the whole range of N/C ratios tested, while it is insensitive only at the restricted region of D around 10^−12^ m^2^/s. *A_0_* is insensitive to D at higher values, and *t_fp_* is insensitive to N/C volume ratios at lower and higher values and to D at lower values. Thus each characterizing parameter possesses different sensitivities to different spatial parameters and different ranges. It should be noted that *τ_p_* and *τ_d_* are strongly sensitive to N/C ratios. Larger N/C ratios result in more prolonged oscillation without changing oscillation frequencies.

## Discussion

We constructed a 3D computational model to see the effect of spatial parameters on the oscillation pattern of nuclear NF-κB and found that N/C ratios, diffusion coefficient, the locus of IκBs synthesis, and nuclear transport altered oscillation patterns. Neither the location nor localization of IκBs transcription or IKK activation altered the oscillation pattern. Thus, there are at least two categories of spatial parameters that alter and do not alter the oscillation pattern of nuclear NF-κB.

When the N/C ratio was increased, the decay time constant *τ_d_* increased in our simulation, indicating the persistent oscillation in larger N/C volume ratios. It is reported that in human cancer patients, both nuclear volume and the N/C ratio are increased [52], [55]. Thus, the oscillation of NF-κB in cancer cells is potentially prolonged. Although there are discussions on the physiological role of persistent oscillation of nuclear NF-κB [64], [65], the persistent oscillation will maintain NF-κB-dependent gene expression [65] and lead to the aberrant gene expression. Our simulation results offer one possible mechanism and explanation for the altered gene expression in cancer cells which have larger N/C ratios.

The change in the nuclear transport altered the oscillation pattern greatly, but differently from changes in the N/C ratio. The change in the number of NPCs can directly alter nuclear transport. In fact, it is reported that in tumor cell lines, Nup88, a component of NPC proteins, was strongly expressed, and its expression level correlated with malignancy [60], [61]. These suggest the increased number of NPCs in cancer cells, and hence the increased nuclear transport. Together with these, our simulation results suggest altered oscillation patterns because of increased nuclear transport in cancer cells

If we changed the spatial localization of IκBs transcription within a nucleus, there was no difference in the oscillation pattern from the control condition (Figure S3). If we changed the localization and the location of IKK activation, there was also no difference from the control conditions (Figure S4). These simulation results should be contrasted with those that have large effects on the oscillation pattern by changes in the N/C ratio, nuclear transport, location of IκBs synthesis, and the diffusion coefficient. If we look at the spatial distributions of nuclear NF-κB and cytoplasmic IKK in our simulation, they are virtually homogeneous (insets in Figure S1A and S4B). These indicate that NF-κB and IKK are well stirred, and this explains the unaltered oscillation pattern by changes in these spatial parameters.

In the present report, we show an altered oscillation pattern of nuclear NF-κB due to changes in spatial parameters, the N/C ratio and nuclear transport that are strongly related to cancer cells. Therefore, it will be important to investigate these spatial parameters in normal and cancer cells.

## Materials and Methods

### Construction of Computational model

Construction of both temporal and 3D models was performed using A-Cell software [66], [67]. Models and all parameters used in the present study can be downloaded from http://www.ims.u-tokyo.ac.jp/mathcancer/A-Cell/index.html.

Our temporal model is basically the same as Hoffmann's model [23] which is shown in Figure 1A. The models for NF-κB activation comprise formation of IKK-IκB-NFκB complexes, degradation of IκBα, nuclear localization of freed NF-κB, NF-κB transcription of IκBα mRNA, IκBs protein synthesis, and nuclear export of IκB-NF-κB complex (Figure S5).

In our 3D model, simulations were performed for a spherical 3D cell with diameter of 50 µm, which was divided into small cubic compartment with identical size enabling reaction-diffusion simulations and evaluation of spatio-temporal pattern of active NF-κB (Figure 1B). There are 62,417 total compartments. Among them the central 8.3% compartments were selected as the nucleus [52], and the most peripheral compartments assigned to the nucleus were selected as the nuclear membrane, which is shown in red in Figure 1B. For cytoplasmic and nuclear membrane compartments, reactions shown in the group of “Cytoplasm” in the temporal A-Cell model were embedded. For nuclear membrane compartments, reactions shown in the groups of “Membrane_in”, “Membrane_out”, and “Protein_synthesis” were embedded. For the nuclear and nuclear membrane compartments, reactions shown in the groups of “Nucleus”, “Transcription”, and “IκBα_transcroption” were embedded.

Additional care was required before simulating the 3D model because there was transportation of proteins and mRNAs by diffusion. This indicates that equilibrium might not be reached when a parameter is changed. Therefore, spatio-temporal equilibrium should be confirmed before starting simulations which is not required for the simulation of the temporal model.

Parameters used in our simulation were modified from Hoffmann's model and are listed in Table S1. The default rate constants for the 3D model should be changed in the 3D model (see Results in main text) and a set of parameters for 3D simulation is listed in Table S2.

### Simulations

Simulation programs written in the c language were automatically generated by A-Cell. Simulations were run on a Linux computer. There are three modes in an automatically generated A-Cell simulation program: the simulation program for a single core, parallelization by openMP for a multi-core CPU, and parallelization by MPI for a supercomputer. For 3D model simulations, the MPI-parallelized simulation program was used to reduce the computational time.

### Analysis

The power spectra for 0–0.4 mHz frequency range were calculated by fast Fourier transformation in Origin 8.5.1 (LightStone). The frequency at the highest peak of a power spectrum was taken as the oscillation frequency for characterizing parameters.

Sensitivity analyses were performed for each characterizing parameter of the oscillation pattern along with the changes in the values of spatial parameters such as N/C ratio and D according to the definition described by the following equation [63]:
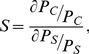
(1)where *P_C_* and *P_S_* are characterizing and spatial parameters, respectively. At each point, the sensitivity was obtained by averaging data from neighbors to the left and right except at the leftmost and rightmost points. Positive and negative sensitivities are shown in reddish and bluish colors, and insensitivity is shown in white in Figure 7.

## Supporting Information

Figure S1 **Homogeneous distribution of nuclear NF-κB in our simulation in the control condition.** (A) Homogeneous distribution at diffusion coefficient of 10^−11^ and 10^−13^ m^2^/s for proteins and mRNA, respectively. The oscillations are plotted at different seven locations from the center to the peripheral compartments of a nucleus. All seven plots completely overlap which is shown by a single line. The spatial homogeneity is also shown in the inset, where the cross-sectional view of nuclear NF-κB is shown. (B) The homogeneous distribution is also seen even at diffusion coefficients for proteins of 10^−13^ m^2^/s. Although there are negligibly small differences among the seven locations, the nuclear distribution of NF-κB is basically homogeneous which is also shown also in the inset. Note that with this small diffusion coefficient, the oscillation pattern was altered greatly (see Main Text).
(TIF)

Figure S2 **Change in the oscillation pattern by the separate change in the inward or outward transport.** Red and blue lines indicate simulation results for changes in inward and outward transport, respectively. (A) Time courses of oscillation for separate changes in inward and outward transport are shown at 1/4, 1/2, 2-folds, and 4-folds changes including control condition (black line). (B) Increases in the outward transport result in the increase in *f*. (C) There is only a small change in *A_0_* by the change in outward transport. A biphasic change is seen by the change in the inward transport. (D) Monotonic decreases in *t_fp_* are seen with increases in the outward transport, and a biphasic change for the inward transport is seen. (E) Data for *τ_p_* were retrieved only within limited regions for the inward and outward transports. Within these regions, *τ_p_* for inward transport shows a biphasic change. (F) Data for *τ_d_* were retrieved also within limited regions. For inward transport, the change in *τ_d_* is biphasic.
(TIF)

Figure S3 **Oscillation pattern with the localized transcription of IκB genes at the center of the nucleus.** There is no difference in the oscillation pattern between the control (thick gray line) and transcription at the center of a nucleus (thin red line).
(TIF)

Figure S4 **Oscillation pattern by the change in the locus of IKK activation.** (A) Tested region (or loci) of IKK activation. Left panel shows the control conditions, and the red compartments in the middle and right panel indicate the locus of IKK activation in the localized cases. (B) No difference in the oscillation pattern is seen by the change in the locus or localization of IKK activation. Thick gray line is the oscillation in control conditions. Thin yellow and blue lines, which overlap perfectly, are in the middle and right panel in A, respectively. Inset shows the homogeneous distribution of IKK in cytoplasm.
(TIF)

Figure S5 **Reactions for IKK, IκBs, NF-κB, and their complexes in the A-Cell temporal model.** All possible interactions shown in Figure 1A were modeled and drawn by A-Cell as shown in the groups, “Cytoplasm” for formation of IKK-IκB-NF-κB complexes, degradation of IκBs, and generation of IκBs-free NF-κB, “Membrane_in” for nuclear localization of freed NF-κB and IκBs, and “IκBα-transcription” for NF-κB transcription of IκBα mRNA, “Protein_synthesis” for IκBs protein synthesis, “Nucleus” for formation of IκB-NF-κB complexes, “Membrane_out” for nuclear export of IκB-NF-κB complex, NF-κB, and IκBs. “Transcription” contains basal transcription of IκBs and their degradation. The reaction parameters are indicated in Table S1 for temporal model and Table S2 for 3D model.
(TIF)

Table S1 **Parameters for the temporal model.**
(PDF)

Table S2 **Parameters for the 3D model.**
(PDF)

Video S1 **Oscillation of nuclear and cytoplasmic NF-κB during simulation period of 10 hrs.** in control conditions. Left, middle, and right movies show oscillations in the whole cell, cytoplasm, and nucleus, respectively. Anti-parallel oscillation between cytoplasm and nucleus is clearly seen in the movie. Virtually no spatial heterogeneity can be seen.
(MP4)
