# Supplementary figures and images for: Roles of Spatial Parameters on the Oscillation of Nuclear NF-κB: Computer Simulations of a 3D Spherical Cell
Source: PLoS One. 2012 Oct 3;7(10):e46911. doi: 10.1371/journal.pone.0046911 (PMC3463570; doi:10.1371/journal.pone.0046911)

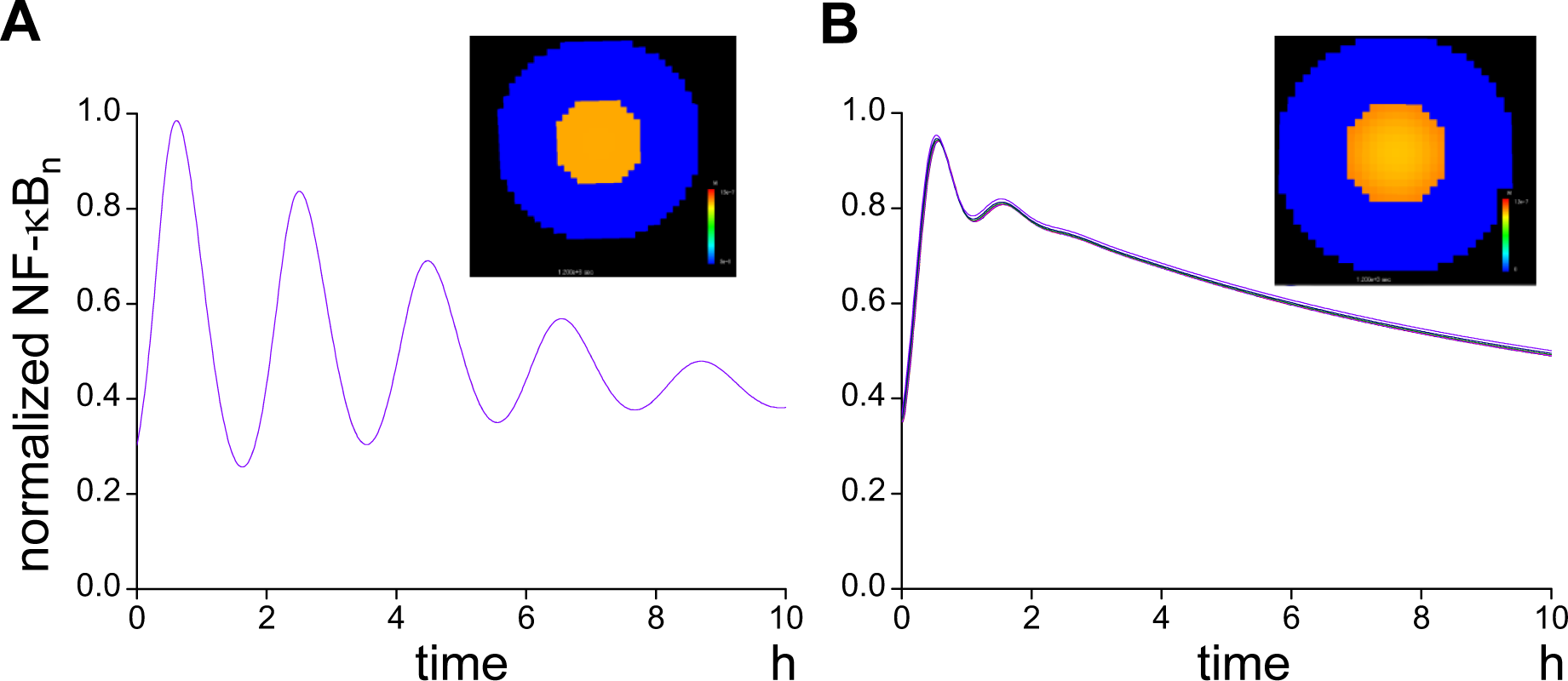

Supplement: Figure S1 — Homogeneous distribution of nuclear NF-κB in our simulation in the control condition. (A) Homogeneous distribution at diffusion coefficient of 10−11 and 10−13 m2/s for proteins and mRNA, respectively. The oscillations are plotted at different seven locations from the center to the peripheral compartments of a nucleus. All seven plots completely overlap which is shown by a single line. The spatial homogeneity is also shown in the inset, where the cross-sectional view of nuclear NF-κB is shown. (B) The homogeneous distribution is also seen even at diffusion coefficients for proteins of 10−13 m2/s. Although there are negligibly small differences among the seven locations, the nuclear distribution of NF-κB is basically homogeneous which is also shown also in the inset. Note that with this small diffusion coefficient, the oscillation pattern was altered greatly (see Main Text). (TIF) [file pone.0046911.s001.tif]

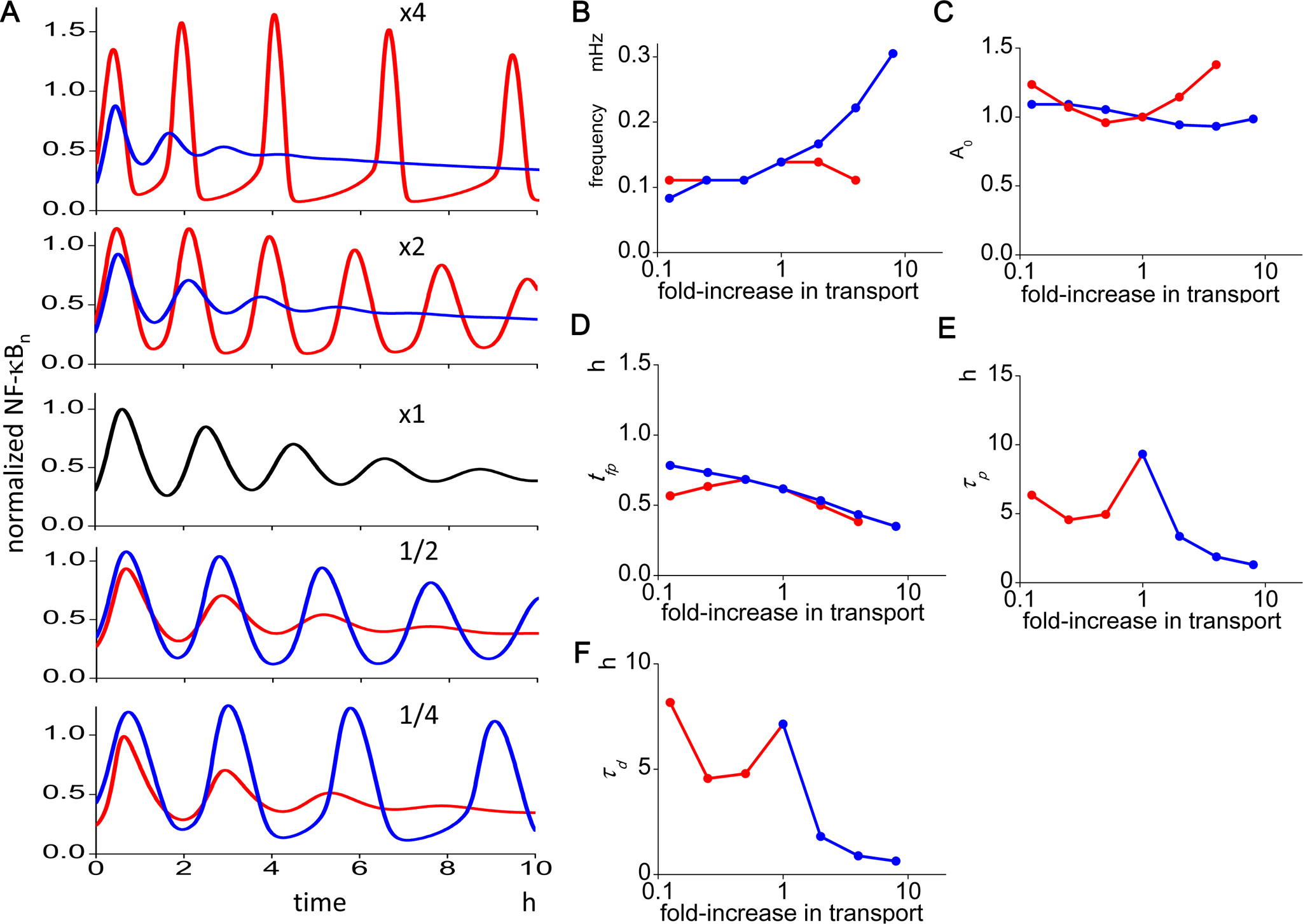

Supplement: Figure S2 — Change in the oscillation pattern by the separate change in the inward or outward transport. Red and blue lines indicate simulation results for changes in inward and outward transport, respectively. (A) Time courses of oscillation for separate changes in inward and outward transport are shown at 1/4, 1/2, 2-folds, and 4-folds changes including control condition (black line). (B) Increases in the outward transport result in the increase in f. (C) There is only a small change in A0 by the change in outward transport. A biphasic change is seen by the change in the inward transport. (D) Monotonic decreases in tfp are seen with increases in the outward transport, and a biphasic change for the inward transport is seen. (E) Data for τp were retrieved only within limited regions for the inward and outward transports. Within these regions, τp for inward transport shows a biphasic change. (F) Data for τd were retrieved also within limited regions. For inward transport, the change in τd is biphasic. (TIF) [file pone.0046911.s002.tif]

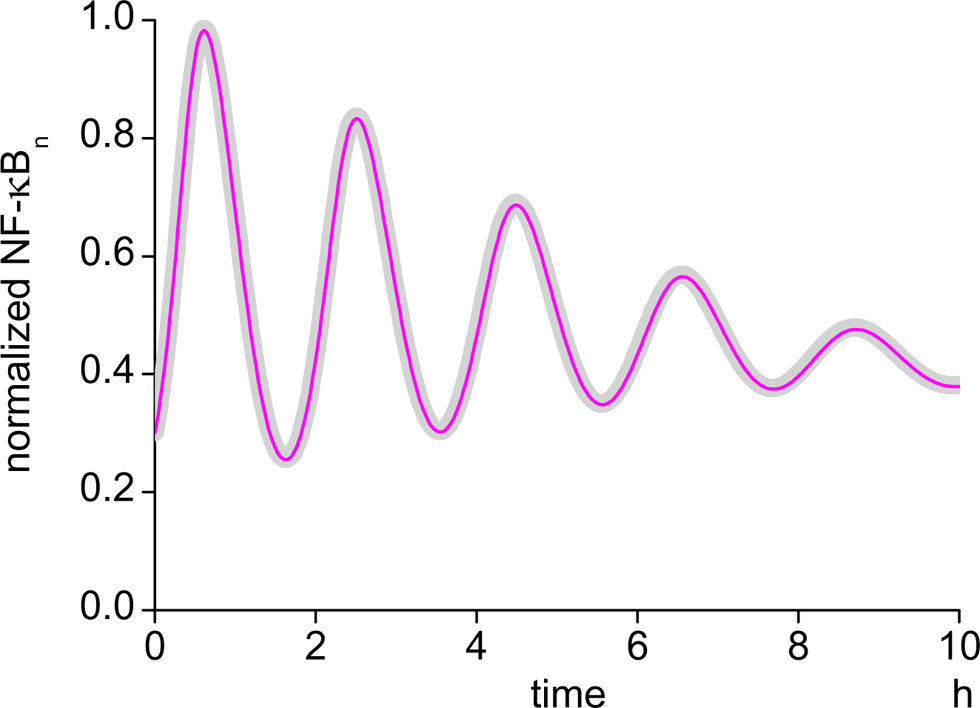

Supplement: Figure S3 — Oscillation pattern with the localized transcription of IκB genes at the center of the nucleus. There is no difference in the oscillation pattern between the control (thick gray line) and transcription at the center of a nucleus (thin red line). (TIF) [file pone.0046911.s003.tif]

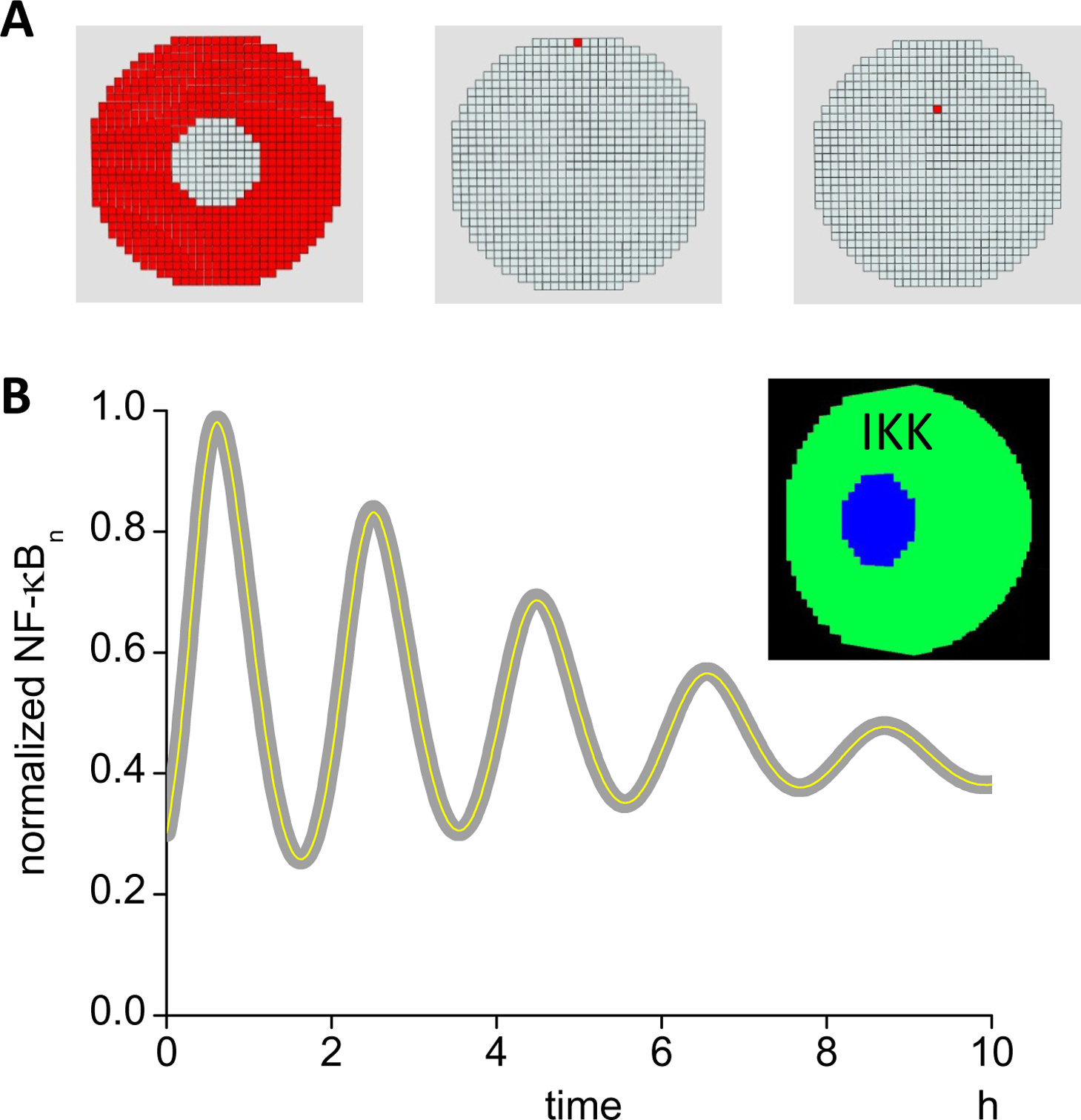

Supplement: Figure S4 — Oscillation pattern by the change in the locus of IKK activation. (A) Tested region (or loci) of IKK activation. Left panel shows the control conditions, and the red compartments in the middle and right panel indicate the locus of IKK activation in the localized cases. (B) No difference in the oscillation pattern is seen by the change in the locus or localization of IKK activation. Thick gray line is the oscillation in control conditions. Thin yellow and blue lines, which overlap perfectly, are in the middle and right panel in A, respectively. Inset shows the homogeneous distribution of IKK in cytoplasm. (TIF) [file pone.0046911.s004.tif]

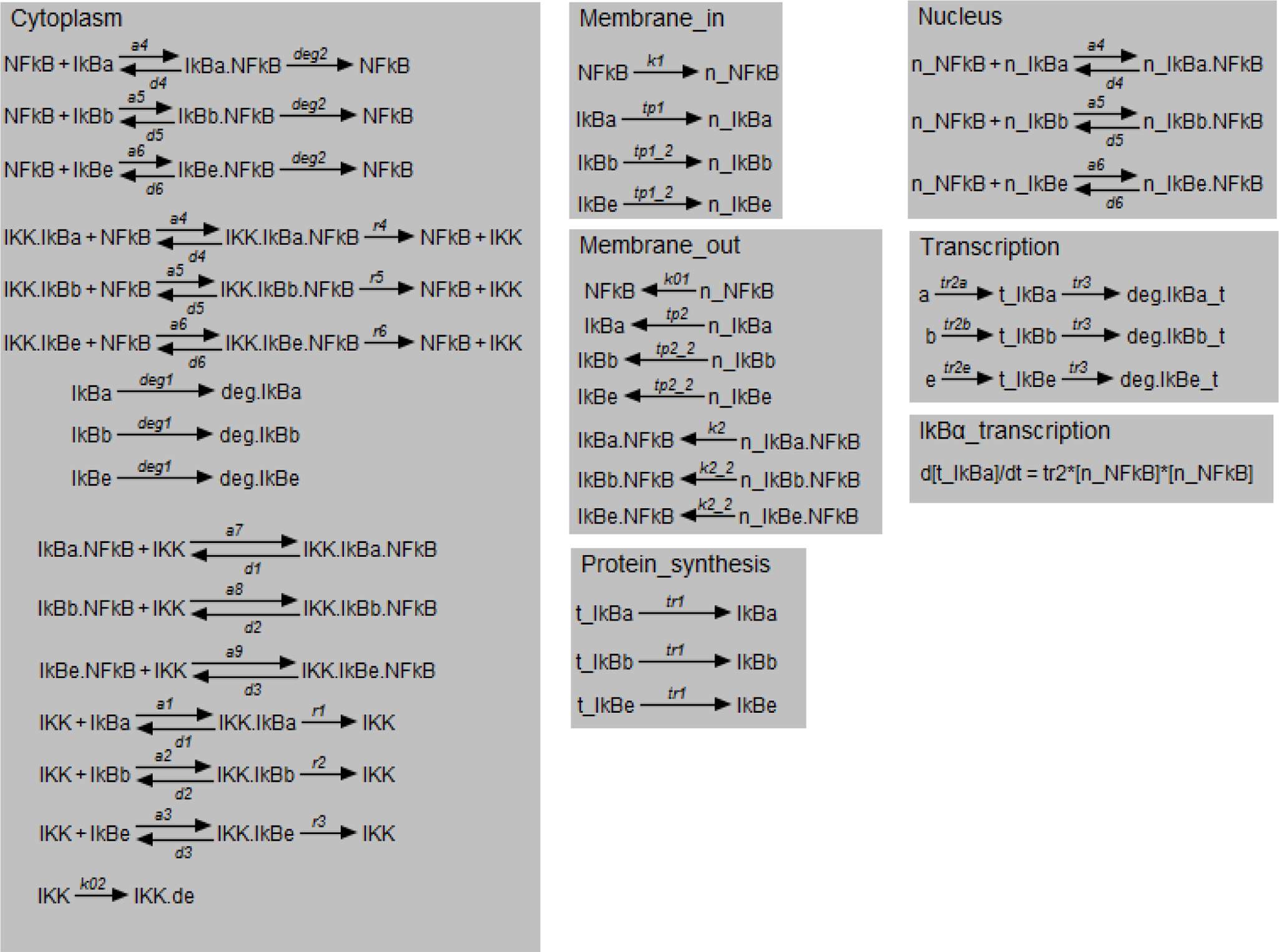

Supplement: Figure S5 — Reactions for IKK, IκBs, NF-κB, and their complexes in the A-Cell temporal model. All possible interactions shown in Figure 1A were modeled and drawn by A-Cell as shown in the groups, “Cytoplasm” for formation of IKK-IκB-NF-κB complexes, degradation of IκBs, and generation of IκBs-free NF-κB, “Membrane_in” for nuclear localization of freed NF-κB and IκBs, and “IκBα-transcription” for NF-κB transcription of IκBα mRNA, “Protein_synthesis” for IκBs protein synthesis, “Nucleus” for formation of IκB-NF-κB complexes, “Membrane_out” for nuclear export of IκB-NF-κB complex, NF-κB, and IκBs. “Transcription” contains basal transcription of IκBs and their degradation. The reaction parameters are indicated in Table S1 for temporal model and Table S2 for 3D model. (TIF) [file pone.0046911.s005.tif]
