# Supplementary material for: Roles of Spatial Parameters on the Oscillation of Nuclear NF-κB: Computer Simulations of a 3D Spherical Cell
Source: PLoS One. 2012 Oct 3;7(10):e46911. doi: 10.1371/journal.pone.0046911 (PMC3463570; doi:10.1371/journal.pone.0046911)
Supplement: Table S1 — Parameters for the temporal model. (PDF) [file pone.0046911.s006.pdf]

**Table S1 Parameters for the temporal model.**

| parameters | values | unit | parameters | values | unit |
|---|---|---|---|---|---|
| a1 | $2.25x10^{+04}$ | /M/s | tp2_2 | $1.00x10^{-04}$ | /s |
| a2 | $6.00x10^{+03}$ | /M/s | tr1 | $4.00x10^{-03}$ | /s |
| a3 | $9.00x10^{+03}$ | /M/s | tr2 | $1.70x10^{+05}$ | /M/s |
| a4 | $5.00x10^{+05}$ | /M/s | a・tr2a | $1.50x10^{-12}$ | M/s |
| a5 | $5.00x10^{+05}$ | /M/s | b・tr2b | $2.30x10^{-13}$ | M/s |
| a6 | $5.00x10^{+05}$ | /M/s | e・tr2e | $1.70x10^{-13}$ | M/s |
| a7 | $1.85x10^{+05}$ | /M/s | tr3 | $2.80x10^{-04}$ | /s |
| a8 | $4.80x10^{+04}$ | /M/s | IKK | $2.00x10^{-09}$ | M |
| a9 | $7.00x10^{+04}$ | /M/s | IKK.IkBa | 0 | M |
| d1 | $1.25x10^{-03}$ | /s | IKK.IkBa.NFkB | 0 | M |
| d2 | $1.75x10^{-03}$ | /s | IKK.IkBb | 0 | M |
| d3 | $1.75x10^{-03}$ | /s | IKK.IkBb.NFkB | 0 | M |
| d4 | $5.00x10^{-04}$ | /s | IKK.IkBe | 0 | M |
| d5 | $5.00x10^{-04}$ | /s | IKK.IkBe.NFkB | 0 | M |
| d6 | $5.00x10^{-04}$ | /s | IkBa | $5.85x10^{-07}$ | M |
| deg1 | $1.00x10^{-04}$ | /s | IkBa.NFkB | $2.95x10^{-07}$ | M |
| deg2 | $2.10x10^{-05}$ | /s | IkBb | $1.97x10^{-09}$ | M |
| k01 | $8.00x10^{-05}$ | /s | IkBb.NFkB | $8.80x10^{-10}$ | M |
| k02 | $8.30x10^{-05}$ | /s | IkBe | $1.45x10^{-09}$ | M |
| k1 | $9.00x10^{-02}$ | /s | IkBe.NFkB | $6.51x10^{-10}$ | M |
| k2 | $1.40x10^{-02}$ | /s | NFkB | $4.07x10^{-10}$ | M |
| k2_2 | $5.60x10^{-03}$ | /s | n_IkBa | $3.64x10^{-09}$ | M |
| r1 | $3.70x10^{-02}$ | /s | n_IkBa.NFkB | $2.49x10^{-09}$ | M |
| r2 | $1.50x10^{-02}$ | /s | n_IkBb | $6.42x10^{-12}$ | M |
| r3 | $2.20x10^{-02}$ | /s | n_IkBb.NFkB | $1.05x10^{-11}$ | M |
| r4 | $1.85x10^{-01}$ | /s | n_IkBe | $4.75x10^{-12}$ | M |
| r5 | $7.50x10^{-02}$ | /s | n_IkBe.NFkB | $7.76x10^{-12}$ | M |
| r6 | $1.10x10^{-01}$ | /s | n_NFkB | $2.00x10^{-08}$ | M |
| tp1 | $6.00x10^{-05}$ | /s | t_IkBa | $8.00x10^{-11}$ | M |
| tp1_2 | $1.50x10^{-04}$ | /s | t_IkBb | $5.37x10^{-11}$ | M |
| tp2 | $4.00x10^{-05}$ | /s | t_IkBe | $3.97x10^{-11}$ | M |
