# Supplementary material for: Roles of Spatial Parameters on the Oscillation of Nuclear NF-κB: Computer Simulations of a 3D Spherical Cell
Source: PLoS One. 2012 Oct 3;7(10):e46911. doi: 10.1371/journal.pone.0046911 (PMC3463570; doi:10.1371/journal.pone.0046911)
Supplement: Table S2 — Parameters for the 3D model. (PDF) [file pone.0046911.s007.pdf]

Table S2 Parameters for the 3D model.

parameters	values	unit	parameters	Values	unit
a1	$4.50x10^{+04}$	/M/s	tp2_2	$1.00x10^{-04}$	/s
a2	$6.00x10^{+03}$	/M/s	tr1	$4.00x10^{-03}$	/s
a3	$9.00x10^{+03}$	/M/s	tr2	$1.70x10^{+05}$	/M/s
a4	$5.00x10^{+05}$	/M/s	a･tr2a	$1.50x10^{-12}$	M/s
a5	$5.00x10^{+05}$	/M/s	b･tr2b	$2.30x10^{-13}$	M/s
a6	$5.00x10^{+05}$	/M/s	e･tr2e	$1.70x10^{-13}$	M/s
a7	$3.70x10^{+05}$	/M/s	tr3	$2.80x10^{-04}$	/s
a8	$4.80x10^{+04}$	/M/s	IKK	$2.00x10^{-09}$	M
a9	$7.00x10^{+04}$	/M/s	IKK.IkBa	0	M
d1	$1.25x10^{-03}$	/s	IKK.IkBa.NFkB	0	M
d2	$1.75x10^{-03}$	/s	IKK.IkBb	0	M
d3	$1.75x10^{-03}$	/s	IKK.IkBb.NFkB	0	M
d4	$5.00x10^{-04}$	/s	IKK.IkBe	0	M
d5	$5.00x10^{-04}$	/s	IKK.IkBe.NFkB	0	M
d6	$5.00x10^{-04}$	/s	IkBa	$2.40x10^{-07}$	M
deg1	$1.00x10^{-04}$	/s	IkBa.NFkB	$3.02x10^{-07}$	M
deg2	$2.10x10^{-05}$	/s	IkBb	$1.34x10^{-10}$	M
k01	$8.00x10^{-04}$	/s	IkBb.NFkB	$1.67x10^{-10}$	M
k02	$8.30x10^{-05}$	/s	IkBe	$9.93x10^{-11}$	M
k1	$9.00x10^{-02}$	/s	IkBe.NFkB	$1.24x10^{-10}$	M
k2	$1.40x10^{-02}$	/s	NFkB	$1.29x10^{-09}$	M
k2_2	$5.60x10^{-02}$	/s	n_IkBa	$1.66x10^{-09}$	M
r1	$3.70x10^{-02}$	/s	n_IkBa.NFkB	$5.13x10^{-09}$	M
r2	$1.50x10^{-02}$	/s	n_IkBb	$4.43x10^{-13}$	M
r3	$2.20x10^{-02}$	/s	n_IkBb.NFkB	$3.59x10^{-13}$	M
r4	$1.85x10^{-01}$	/s	n_IkBe	$3.27x10^{-13}$	M
r5	$7.50x10^{-02}$	/s	n_IkBe.NFkB	$2.65x10^{-13}$	M
r6	$1.10x10^{-01}$	/s	n_NFkB	$4.92x10^{-08}$	M
tp1	$3.00x10^{-04}$	/s	t_IkBa	$1.73x10^{-07}$	M
tp1_2	$1.50x10^{-04}$	/s	t_IkBb	$9.60x10^{-11}$	M
tp2	$2.00x10^{-04}$	/s	t_IkBe	$7.10x10^{-11}$	M
